# Loss of FBXW7-mediated degradation of BRAF elicits resistance to BET inhibitors in adult T cell leukemia cells

**DOI:** 10.1186/s12943-020-01254-x

**Published:** 2020-09-09

**Authors:** Chien-Hung Yeh, Marcia Bellon, Fang Wang, Hong Zhang, Liwu Fu, Christophe Nicot

**Affiliations:** 1 Department of Pathology and Laboratory Medicine, University of Kansas Medical Center, 3901 Rainbow Boulevard, Kansas City, KS 66160 USA; 2 State Key Laboratory of Oncology in South China, Guangdong Esophageal Cancer Institute, Sun Yat-sen University Cancer Center, Guangzhou, China

## Abstract

**Background:**

Human T cell leukemia virus type 1 (HTLV-1)-associated adult T cell leukemia (ATL) has a very poor prognosis with a median survival of 8 months and a 4-year overall survival of 11% for acute ATL. Present treatment options are limited and there is no curative therapy for ATL. Ubiquitin ligase FBXW7 is commonly mutated or functionally inactivated in human cancers. Consistent with the notion that FBXW7 controls the degradation of many oncoproteins, loss of FBXW7 has been linked to increased drug resistance or sensitivity in cancer cells.

**Method:**

In this study, we have characterized FBXW7 mutants previously identified in HTLV-I-infected ATL patient samples. TET-inducible ATL cells carrying wild type or mutated FBXW7 were analyzed for target degradation and for drug sensitivity.

**Results:**

Our results demonstrate that mutations in FBXW7 can selectively disrupt ubiquitination and proteasome degradation of target proteins: c-MYC, cyclin E and MCL1. Both c-MYC and MYCN were highly expressed in uncultured ATL patient’s samples and ATL-derived cell lines; and ATL cells demonstrated sensitivity to BET inhibitors in vitro and in vivo. High-throughput reverse phase protein array revealed BRAF as a novel target of FBXW7 and further experiments showed that mutations in FBXW7 preventing degradation of BRAF provided resistance to BET inhibitors. In contrast to R465, hot spot FBXW7 mutations at R505C retained degradation of BRAF but not NOTCH1, c-MYC, cyclin E, or MCL1. Finally, a combination therapy using BET inhibitors along with a BRAF or an ERK inhibitor prevented tumor cell resistance and growth.

**Conclusion:**

Our results suggest that FBXW7 status may play an important role in drug therapy resistance of cancer cells. Genetic characterization of FBXW7 may be one factor included in future personalized cancer treatment identification.

## Introduction

The FBXW7 ubiquitin ligase and tumor suppressor is known to target many oncoproteins, such as NOTCH1, AURKA, mTOR, c-MYC, cyclin E and MCL1 for proteasome-mediated degradation [1, 2]. Phosphorylation of the conserved FBXW7 phosphodegron motifs on the substrates are essential for FBXW7 to interact with and to target substrates for degradation. FBXW7 is the most commonly inactivated ubiquitin-proteasome system protein in human cancer. The relative low frequency of single-FBXW7 substrate CPD mutations compared with FBXW7 mutations implies the requirement for deregulation of several oncoproteins in FBXW7-related tumorigenesis [3]. In addition to genetic inactivation, epigenetic mechanisms have been reported to decrease FBXW7 expression. MicroRNA miR-223 is highly expressed in ATL patient samples; and miR-223 can directly target FBXW7 [4, 5]. Importantly, several studies demonstrate that the miR-223/FBXW7 axis regulates cisplatin, doxorubicin and trastuzumab resistance. Additional studies show that loss-of-function of FBXW7 in lung cancer cells confer resistance to gefitinib, cetuximab or panitumumab. In colorectal cancer (CRC), FBXW7 loss confers resistance to oxaliplatin and cisplatin chemotherapeutic agents, while CRC cell lines harboring FBXW7 mutations or deletions are more sensitive to rapamycin treatment. Loss of FBXW7 also mediates increased resistance of CRC cells towards taxol and vincristine that can be overcome by inhibiting MCL1 [6]. The fact that FBXW7 controls many distinct signaling pathways makes it an attractive target for therapeutic intervention.

Human T-cell leukemia virus type 1 (HTLV-1), infects more than 20 million people worldwide; and is the causative agent of adult T-cell leukemia (ATL) and HTLV-1-associated myelopathy/tropical spastic paraparesis (HAM/TSP) [7–9]. Many of the FBXW7 substrates including NOTCH1, c-MYC, cyclin E and MCL1 have been reported to play a role in HTLV-1-mediated T cell growth, survival and/or transformation. In our previous studies, we reported PEST domain NOTCH1 mutations in 30% of ATL patients resulting in increased NOTCH1 stability and reduced FBXW7-mediated degradation [10]. The biological significance of NOTCH signaling in ATL was demonstrated by blockade of NOTCH1 signaling with either dominant negative MALM1 or gamma secretase inhibitor, which significantly reduced ATL tumor growth in vitro and in a xenograft mouse model of ATL [10]. Since NOTCH1 was activated even in the absence of genetic mutations in ATL cells we investigated the expression of FBXW7. Our results showed that FBXW7 expression was down-regulated in ATL patients’ cells and mutated in about 25% of primary ATL patient samples. FBXW7 loss-of-function led to an increase in ATL cell proliferation and transformation both in in vitro and in vivo xenograph models [11]. The inactivation of checkpoints that control G1/S progression is frequent in HTLV-1 infected cells. The viral oncoprotein Tax has been shown to upregulate c-MYC expression through the activation of the NF-κB signaling pathway [12]. Increased c-MYC expression stimulates cellular proliferation and hTERT expression thereby facilitating T cell immortalization. Additional studies have also shown that tumors derived from Tax transgenic mice express high levels of c-MYC [13]. Most HTLV-1 transformed cells require c-MYC signaling and silencing of c-MYC expression impairs the growth of HTLV-1-transformed human T-cell lines [14]. Abnormal cyclin E expression is observed in many cancers and has been linked to increased genomic instability [15–17]. Expression of viral Tax in human T cells induces the expression of cyclin E and CDK2 [18]. MCL-1 belongs to the Bcl-2 family and regulates apoptosis in normal and cancer cells [19]. MCL-1 is highly expressed in leukemia [19] and the expression of MCL-1 is correlated with chemotherapy response [20]. Expression of Tax in CD4+ T cells causes hyper-proliferation and immortalization with increased expression of MCL-1 [21]. Importantly, FBXW7 deletion or loss-of-function mutations from patient-derived cancer cells impair the MCL-1 degradation and result in resistance to chemotherapy drugs [6, 22].

In this study, we found that c-MYC and MYCN are highly expressed in freshly isolated uncultured ATL samples. Targeting c-MYC with BET inhibitors JQ1 or OTX-015 significantly affected growth of ATL cell lines, which were less sensitive to cyclin E or MCL1 inhibitors. Using a high-throughput reverse phase protein array we found that FBXW7 targets BRAF for degradation. Moreover, mutations in FBXW7 that prevented BRAF degradation were associated with resistance to BET inhibitors. Our results suggest that BRAF/ERK signaling is an important mediator of FBXW7 mutant-mediated resistance to BET inhibitors. Although it is challenging to target specific FBXW7 mutants, the deregulation of distinct downstream targets as a result of FBXW7 mutations reveals unique cancer dependencies and provides opportunities for targeted personalized therapeutic intervention.

## Methods

### Cell Lines

DMEM (Invitrogen) with 10% FBS (Atlanta Biologicals) was used for 293 T cell culture and maintained in 5% CO2 at 37 °C. The ATL patient-derived cell lines MT1, ATL25, ED-40515(−), Tl-Om1, and ATLT, along with the HTLV-I cell line, MT4, were cultured in RPMI-1640 (Invitrogen) with 10% FBS in 5% CO2 at 37 °C. The ATL patient-derived cell lines, KOB, KK1, ATL55T, and ATL43T, were cultured in RPMI-1640 with 20% FBS and 50 U/ml IL-2. Cell proliferation was analyzed by XTT assays (Trevigen) or by microscopic cell counts at different times after drug treatment as indicated in the figure legends. JQ1, OTX015, and AG490 were purchased from Cayman Chemical. Dinaciclib (SCH727965), PLX8394, Sorafenib, and Ulixertinib (BVD-523) selective inhibitors were purchased from Selleckchem.

### Plasmids and Transfections

FBXW7 mutants were made by QuikChange Site-Directed Mutagenesis Kit (Agilent) following manufacturer’s protocol. The pTripZ vector (Thermo) was used to generate high-titer Tet-inducible FBXW7 lentivirus. Polyfect (Qiagen) and Calcium Phosphate Transfection Kit (Invitrogen) were used for 293 T transfection and lentivirus production, respectively. MT1 cells were infected with FBXW7 wild-type or mutant virus and maintained in culture with RPMI-1640 with 10% FBS (TET-Tested; Atlanta Biologicals) with puromycin. Induction of FBXW7 was carried out in the presence of doxycycline for various times.

### Western Blots and Co-Immunoprecipitation (co-IP)

FBXW7 substrates conjugated with Myc tag (cyclin E and BRAF) were analyzed by anti-Myc (9E10; Roche), whereas anti-HA (3F10) (Roche) was used to detect FBXW7 substrates conjugated with HA tag (c-MYC and MCL1). Anti-Flag M2(Sigma #F3165) was used to for FBXW7 and mTOR (Addgene #26603) detection. Endogenous expression of proteins was performed using antibodies for CyclinE (Santa-Cruz #sc-198), Mcl-1 (Santa-Cruz #sc-12,756), c-myc (9E10), FBXW7 (Proteintech #28424–1-AP), BRAF (Santa-cruz #sc-5284), p-MEK-1/2 (Calbiochem), p-ERK-1/2, and STAT3 (Santa-Cruz #sc-482). All western blots were normalized to Actin expression (Santa-Cruz #sc-8432). For co-immunoprecipitations, 293 T cells were co-transfected with tagged FBXW7 and its substrate(s) for 48 h. Cells were lysed in NP-40 lysis buffer (50 mM KCl, 10 mM Tris-HCl pH 8.8, 5 mM MgCl2 and 0.65% NP-40), immunoprecipitated with appropriate tagged antibodies, and immunoblotted by the indicated antibody as described in the figure legends.

### Immunohistochemistry (IHC)

Cells resuspended at 1.10^6 per ml in 4% formaldehyde and fixed by incubating for 20 min at room temperature. Cells were washed twice with PBS and resuspended in PBS and spotted on slides. Cells were permeabilized with 0.5%Triton-X-100 for 20 min rinsed with PBS for 2 times and endogenous peroxidase was quenched by 20-min incubation in 3% hydrogen peroxide. Cells were washed with PBS and blocked with serum blocking reagent for 15 min before incubation with primary antibodies overnight at 4 °C. The following day, slides were washed with PBS and incubated with HRP-labeled anti-rabbit/mouse secondary antibody for 30 min, washed with PBS, and 200 μl DAB substrate solution was added to the cells. Slides were washed with PBS, counterstained by hematoxylin and washed with water for 10 min. Dehydration was performed by four incubations with alcohol (75, 85, 95 and 100%) for 5 min each. Slides were incubated 3 times with xylene and coverslip using mounting solution.

### Ubiquitination Assays

293 T cells were co-transfected with tagged FBXW7 substrates, Flag-tagged FBXW7 and HA-tagged Ub (K48) for 48 h. Six hours before harvest, cells were treated with 10 μM MG132 to inhibit proteasome-mediated degradation. Cells were harvested in NP-40 lysis buffer containing N-ethylmaleimide, iodoacetamide, and EDTA. The lysates were then immunoprecipitated and immunoblotted.

### Drug treatment and Reverse-phase protein array (RPPA) analysis

FBXW7 wild-type (WT) or FBXW7 mutant, S462P, MT1 cells (seeded at 0.25 × 10^6 cells/ml) were induced with 2 μg/ml doxycycline for 24 h before drug treatment. After 72 h treatment with JQ1, Tet-inducible FBXW7 WT and S462P-expressing cells were collected and sent for RPPA analysis according to protocols from RPPA Core Facility, MD Anderson Cancer center.

### Xenograft Model

Female athymic nude mice (BALB/c-nu/nu), 4 to 5 weeks old and weighing 18 to 22 g were used for the ATL xenograft model. Before injection of MT1 ATL cells, mice were given 2 mg/mL doxycycline (MCE, HY-N0565B) in the drinking water with 2% sucrose for 7 days. The water was thereafter changed every day. Total 200 μl mixtures (volume ratio = 1:1) of matrigel matrix (Corning® Matrigel® Matrix, 354,234) and suspension of 2 × 10^7 WT FBXW7 MT1 cells were injected subcutaneously. The mice were randomized into two groups after the tumors reached a mean volume of about 50 mm3, and then treated with (+)-JQ1 (50 mg/kg, i.p., q3d, MCE, HY-13030) or vehicle. The body weight and two perpendicular tumor diameters (A and B) were measured every 3 days, and the tumor volume (V) was calculated according to the following formula: V = (π/6 × width2 × length). Tumor growth was plotted as tumor volume versus time since inoculation. At termination, tumors were excised, weighed, and photographed. The ratio of growth inhibition (IR) was calculated according to the following formula: “IR” = 1-(Mean tumor weight of experimental group)/(Mean tumor weight of control group) × 100%. All animal care and experimental procedures were approved by the Ethics Committee for Animal Experimentation (Center of Experimental Animals, Sun Yat-Sen University, China).

### Gene Array and Real-time PCR

Total RNA from healthy, non-HTLV-I infected PBMCs and uncultured, ATL patient PBMCs were extracted using TRIzol RNA extraction reagent (Invitrogen) and treated with DNaseI. DNA-free RNA was reverse transcribed using the RNA-to-cDNA kit (Invitrogen). Total cDNA was amplified using iTaq Universal SYBR Green Supermix (Biorad) on the StepOnePlus Real-time PCR instrument (Applied Biosystems). Primers used for this study include: GAPDH, F: 5′-GAAGGTGAAGGTCGGAGTC − 3′ and R: 5′-GAAGATGGTGATGGGATTTC-3′; RPX3, F: 5′-ATCCCGTGGAGACTCCTCAA-3′ and R: 5′-AACACGTAGACTGGGTATCC-3′; HBZ, F: 5′-CGGCCTCAGGGCTGTTTC-3′ and R: 5′-GCGGCTTTCCTCTTCTAAGGA; cmyc, F: 5′-CGTCTCCACACATCAGCACAA-3′ and R: 5′-TCTTGGCAGCAGGATAGTCCTT-3′, and nmyc, F: 5′-CACAAGGCCCTCAGTACCTC-3′ and R: 5′-ACCACGTCGATTTCTTCCTC-3′. Results were normalized to GAPDH expression. The MYC gene array was carried out using Human MYC Targets RT^2^ Profiler PCR array (Qiagen). Briefly, 1μg of RNA from healthy, non-HTLV-I infected PBMCs or uncultured ATL PBMCs were treated with DNaseI and reverse transcribed into cDNA with the RNA-to-cDNA kit. Equal amounts of cDNA were applied to each well; and gene expression was detected using iTaq Universal SYBR Green Supermix on the StepOnePlus Real-time PCR instrument. Data was analyzed using Qiagen data analysis software.

### Statistical methods

Experiments presented in figures were performed 2 or 3 times each run in duplicate. Representative results were shown in the final Figs. *P* values were calculated using two-tailed distribution Student’s t test on paired or unpaired data sets. In the figures, an asterisk denotes a *p* value < 0.05. In addition, as needed to determine the strength of the data relationship, correlation analysis was performed by using Pearson’s correlation. The Pearson’s correlation coefficient, coefficient of determination, r and *p* values are reported in the figure and/or figure legends.

## Results

### Up-regulation of c-MYC and MYCN in freshly isolated uncultured ATL samples

**Fig. 1:**
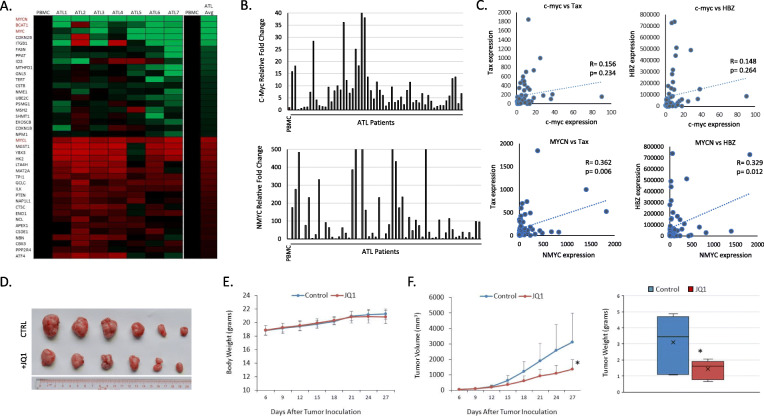
Activation of the MYC pathway in ATL patients. **a** MYC RT profiler arrays were performed on normal, non-HTLV-I infected, PBMCs and 7 PBMCs from uncultured, ATL patients’ samples. The top 20 up-regulated and down-regulated genes, compared to PBMCs, are highlighted. Fold change is compared to PBMCs. ATL patients, ATL2 (m425), ATL3 (m462), and ATL4 (m468) have mutations in FBXW7. ATL1 is wild-type FBXW7. **b** c-MYC and NMYC are highly expressed in ATL patients. Real-time PCR analysis of c-MYC and NMYC expression in ATL patients (*n* = 57) were performed in duplicates. Fold change is compared to normal PBMCs and patient samples are normalized to GAPDH expression. **c** c-MYC and NMYC do not significantly correlate with HTLV-I viral genes. Real-time PCR expression was determined for Tax, HBZ, c-MYC, and NMYC in uncultured ATL patients’ samples. Samples were normalized to GAPDH expression. Gene expression values of Tax and HBZ were plotted against gene expression values of c-MYC and NMYC. Correlations between MYC genes and Tax/HBZ were determined by Pearson Correlation Coefficient. *p*-values are indicated. **d** In vivo inhibition of MYC activity inhibits ATL cell tumor growth in a mouse xenograph model. MT1 cells stably transfected with a TET-inducible FBXW7 expression vector were injected into BALB/c-nu/nu mice. The mice were randomized into two groups after the tumors reached a mean volume of about 50 mm3, and then treated with vehicle or BET inhibitor JQ1. After 4 weeks tumors were surgically excised, weighed, and photographed. **e** The body weight was measured every 3 days. * is *P* value of < 0.05. **f** Tumors volume and tumors weight were measured in JQ1 treated animals and compared to controls. * is P value of < 0.05

Deregulation of c-MYC has been extensively investigated in many cancer types including leukemia and lymphoma but little is known about the expression and role of c-MYC in ATL lines and uncultured ATL cells. To investigate the c-MYC signaling pathway in ATL, mRNAs were extracted from uncultured ATL primary samples and a negative control not infected with HTLV-I and subjected to c-MYC microarray analyses. Our results demonstrate high levels of both c-MYC and MYCN expression in all ATL patients’ samples (Fig. 1a). Further real time RT-PCR analyses from 60 uncultured ATL samples confirmed amplification of c-MYC and MYCN expression (Fig. 1b). It is well known that tumor suppressor p53 is a potent transcriptional repressor of c-MYC, and although p53 is rarely mutated in ATLL cells, studies have shown that its transcriptional activities are inhibited in ATL cells. In addition, FBXW7, negative regulator of c-MYC, is frequently mutated in acute ATL patients (25%) resulting in stabilization of NOTCH1 and NOTCH1-dependent activation of c-MYC expression. These observations suggest that c-MYC activation may play an important role in ATL pathogenesis. Consistent with this notion, studies have shown that HTLV-I transformed cells are addicted to MYC pathway activation inasmuch as blockade of MYC-dependent transcription with JQ1 inhibitor triggers an apoptotic signal in Tax-expressing cell lines [23]. We next investigated whether high levels of c-MYC and MYCN expression in ATL cells correlated with the expression viral proteins Tax or HBZ. Although Tax has been shown to stimulate the c-MYC promoter through activation of the NF-kB pathway, we did not find any correlation between viral Tax or HBZ expression and c-MYC or MYCN levels in ATL cells (Fig. 1c). In contrast to c-MYC and MYCN, MYCL, a paralog of MYCN, was downregulated in ATL patient samples. We next investigated the importance of the c-MYC signaling pathway for ATL cells proliferation and tumor formation in vivo. To this end ATL cells, MT1, stably transfected with a TET-inducible FBXW7 expression vector were injected into BALB/c-nu/nu mice. The mice were randomized into two groups after the tumors reached a mean volume of about 50 mm3, and then treated with vehicle or BET inhibitor JQ1. After 4 weeks tumors were surgically excised, weighed, and photographed (Fig. 1d). The body weight was measured every 3 days and remained similar between vehicle or JQ1 treated mice, as demonstrated by an absence of general toxicity (Fig. 1e). Tumors volume and tumors weight were significantly smaller in JQ1 treated animals compared to controls (Fig. 1f). Together our data demonstrate in vivo dependency of ATL cells for c-MYC signaling.

Our microarray data also demonstrated high expression of the BCAA transaminase 1 (BCAT1) gene known to transfer α-amino groups from branched-chain amino acids to α-ketoglutarate. Increased levels of BCAT1, a target of c-MYC, has been previously associated with more aggressive cancers [24]. Depletion of BCAT1 reduces survival of AML cells in vitro as well as in vivo. BCAT1 overexpression decreased intracellular αKG levels and caused DNA hypermethylation through inhibition of TET2, mimicking the effects of IDH mutations in AML [25]. These observations may be relevant to ATL pathogenesis since we have previously reported a high frequency of TET2 missense mutations (25%) and loss of heterozygosity (LOH) of TET2 (10%) in ATL patients with the acute type [26]. Moreover, an increased genome methylation has been reported during progression of ATL diseases [27]. Therefore, a possible role for BCAT1 in ATL pathogenesis warrants further investigations.

### FBXW7 mutations found in ATL patients selectively affect proteasome degradation of c-MYC, Cyclin E, and MCL1

**Fig. 2:**
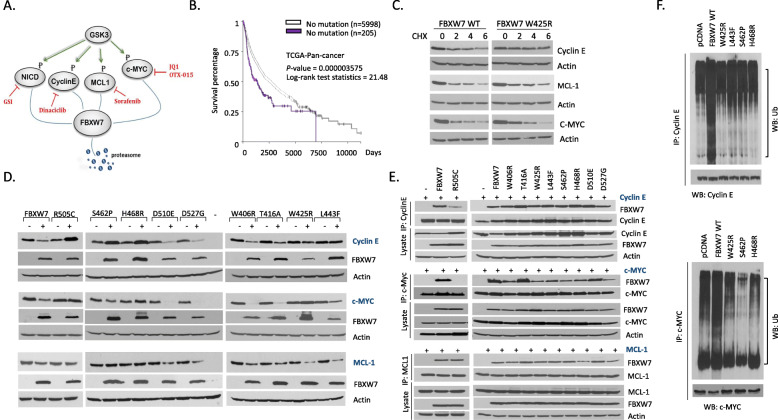
FBXW7 mutants selectively target c-Myc, CyclinE and MCL1 for degradation. **a** GSK3 phosphates NOTCH1, cyclin E, MCL1 and c-MYC at specific phosphodegrons. The phosphorylation is essential for FBXW7-mediated proteasome degradation. Oncoprotein specific inhibitors are indicated the Figs. **b** FBXW7 mutations are correlated with poor prognosis in TCGA pan-cancer analysis. **c** FBXW7 W425R increased the half-life of cyclin E and c-MYC but had no effect on MCL1 half-life. The effect of FBXW7 on the half-life of oncoproteins was determined by Western blot after cells treated with 100 μg/mL cycloheximide (CHX) for 0, 2, 4, and 6 h. **d** FBXW7 mutations impaired the degradation of oncoproteins. FBXW7-mediated degradation of oncoproteins was determined by Western blot. FBXW7 WT and R505C were used as positive and negative controls, respectively. **e** The interaction between FBXW7 and oncoproteins was analyzed by co-immunoprecipitation. 293 T cells co-transfected with FBXW7 and oncoprotein were lysed and immunoprecipitated with oncoprotein. Western blot was used to analyze the interaction between FBXW7 and oncoproteins. **f** FBXW7-mediated ubiquitination of cyclin E and c-MYC was deficient by FBXW7 mutations. 293 T cells were co-transfected with indicated FBXW7, K48-Ub and cyclin E (upper panel) or c-MYC (lower panel). Cells were treated with MG132 for 6 h before harvest. Immunoprecipitated cyclin E or c-MYC and Western blot Ub showed the ubiquitination level of cyclin E (upper panel) and c-MYC (lower panel). **g** Table summarizing FBXW7 wild-type and FBXW7 mutants

We have previously reported novel mutations in the WD40 domain of FBXW7 preventing degradation of NOTCH1 in ATL cells and decreased in vivo tumor growth by blockade of NOTCH1-dependent growth of ATL cells [10, 11]. However, the effect of these mutations on other well-known targets of FBXW7 (Fig. 2a) is unknown. Some studies have suggested that hot spot mutations at specific arginine residues R505, R465 and R479 result in complete inactivation and complete loss of FBXW7 functions [28]. To our knowledge FBXW7 mutations specifically affecting some targets and not others have not been reported except for our previous study on NOTCH1. We have previously discovered that FBXW7 mutants D510E and D527G are able to degrade cyclin E, c-MYC and MCL1 just as well as their wild type counterpart but these mutants were unable to degrade NOTCH1. Therefore, further characterization of FBXW7 mutations is critically important to fully understand the mechanisms that dictate FBXW7 target interactions and degradation. This is further exemplified by the fact that some mutations found in ATL patients have been detected in other human cancers (S462F, W425C, L443F and D527G); and Kaplan Meier FBXW7 somatic mutation TCGA-Pan-cancer data (http://xena.ucsc.edu/) analyses suggest that mutations in the FBXW7 gene adversely affect survival of cancer patients (Fig. 2b). We previously reported that some FBXW7 mutations prevent the degradation of NOTCH1 [11]. We then investigated the half-life of additional targets of FBXW7 in cells transfected with wild type or W425R FBXW7 mutant. Thirty-six hours following transfection cells were treated with cyclohexamide (CHX) to prevent de novo protein synthesis. Western blot analyses confirmed an extended half-life and reduced turnover of Cyclin E, MCL1 and c-MYC in cells carrying W425R (Fig. 2c).

FBXW7 wild type, the FBXW7 R505C reportedly dead-mutant, and additional FBXW7 mutants (S462P, H468R, D510E, D527G, W605R, T416A, W425R, and L443F) previously isolated from ATL cells were transiently transfected along with tagged-expression vectors for cyclin E, c-MYC or MCL1. The ability of transfected FBXW7 and mutants to degrade targets were assessed by western blot analyses (Fig. 2d). As expected, wild type FBXW7 was able to induce degradation of cyclin E, c-MYC and MCL1 while R505C was not. In contrast to wild type FBXW7, our studies suggested that R505C weakly interacted with cyclin E and interacted with MCL1 as well as wild-type, but it did not bind c-MYC (Fig. 2e). These data are similar to those we have previously reported regarding NOTCH1 and ATL FBXW7 mutants [10, 11], and suggest that a simple lack of interaction is not sufficient to explain FBXW7 phenotypes. Unexpectedly, western blot analyses revealed that different ATL FBXW7 mutants had differential abilities to degrade cyclin E, c-MYC and MCL1 (Fig. 2e). FBXW7 S462P and H468R mutations present a phenotype similar to that of R505C and could not induce degradation of any targets. Surprisingly FBXW7 W425R and L443F mutants could only induce degradation of MCL1. FBXW7 W406R and T416A were similar to wild type FBXW7, while D510E and D527G could degrade all targets with the exception of NOTCH1 [10]. Lack of degradation observed for some of FBXW7 mutations was not due to lack of interactions as demonstrated by co-immunoprecipitation assays (Fig. 2e). Rather defects in the ability to add ubiquitin to the target protein seem to be the determining factor as demonstrated by co-immunoprecipitation assays (Fig. 2f). Table 1 summarizes the different phenotypes of FBXW7 mutations identified in ATL patients’ cells (Fig. 2g).

**Table 1:** Phenotypic characterization of FBXW7 mutants

	NICD	Cyclin E	c-Myc	MCL-1	BRAF
FBXW7 WT	**+**	**+**	**+**	**+**	**+**
S462P	-	-	-	-	-
H468R	-	-	-	-	-
W425R	-	-	-	**+**	-
L443F	-	-	- **/+**	**+**	+
W406R / T416A	**+**	**+**	**+**	**+**	+
D510E /D527G	-	**+**	**+**	**+**	+
R505C	-	-	-	-	+
R465C	-	-	-	-	

### ATL cells are sensitive to BET inhibitors; but mutations in FBXW7 provide tumor cell resistance

**Fig. 3:**
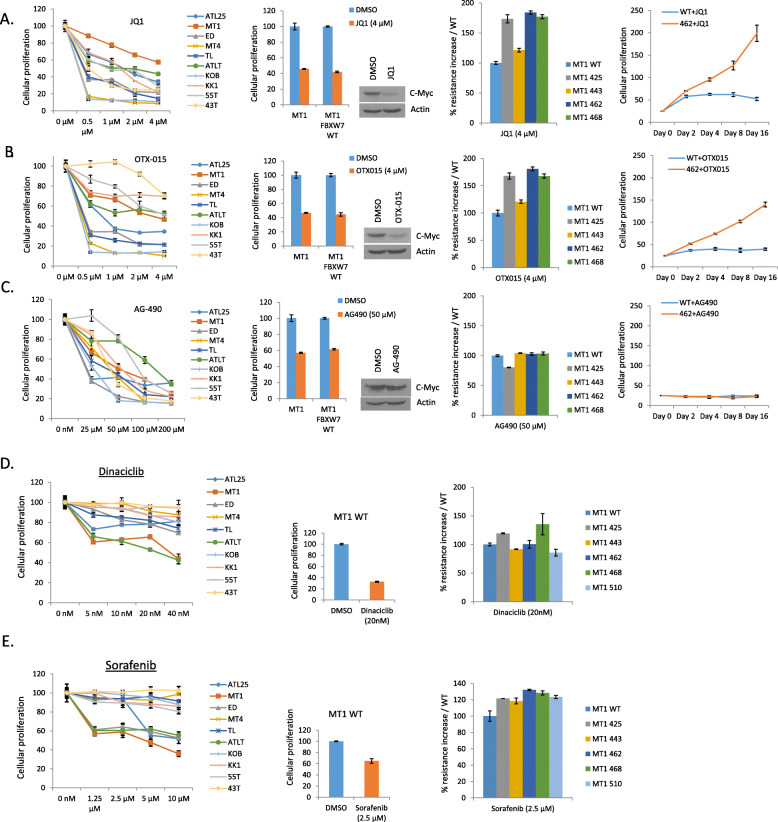
FBXW7 mutations caused resistance to c-MYC inhibitors. JQ1 (c-MYC inhibitor) (**a**), OTX-015 (c-MYC inhibitor) (**b**) and AG-490 (JAK2 inhibitor) (**c**) reduced HTLV-I/ATL-lines proliferation in a dose-dependent manner. Cells were treated with the indicated concentration of inhibitor or dimethyl sulfoxide (DMSO) for 72 h. Each cell line was treated at least twice for standard deviation. Cell proliferation was analyzed by XTT assay (Roche). Overexpression of FBXW7 WT in MT1 didn’t affect JQ1 (**a**), OTX-015 (**b**) and AG-490 (**c**) sensitivity. Western blot analysis showed the reduced expression of c-MYC by JQ1 and OTX-015. Expression of FBXW7 mutants caused resistance to JQ1 (**a**) and OXT-015 (**b**) treatment, but not AG-490 (**c**) treatment. MT1 cells expressing the indicated FBXW7 wild-type or FBXXW7 mutants were treated with JQ1/OTX-015/AG-490 or dimethyl sulfoxide (DMSO). Cell proliferation was analyzed by XTT assay (Roche). Dinaciclib (cyclin E inhibitor) (**d**) and Sorafenib (MCL1 inhibitor) (**e**) reduced HTLV-I/ATL-lines proliferation in a dose-dependent manner. Cells were treated with the indicated concentration of inhibitor or dimethyl sulfoxide (DMSO) for 72 h. Each cell line was treated at least twice for standard deviation. Cell proliferation was analyzed by XTT assay (Roche). Expression of FBXW7 mutants caused resistance to Dinaciclib (cyclin E inhibitor) (**a**) and Sorafenib (MCL1 inhibitor) (**b**) treatment. MT1 cells expressing indicated FBXW7 wild-type or FBXW7 mutants were treated with Dinaciclib/Sorafenib or dimethyl sulfoxide (DMSO) for 72 h. Cell proliferation was analyzed by XTT assay (Roche)

To investigate the dependence of ATL cells on constitutive activation of the MYC pathway, we tested two different bromodomain and extra-terminal (BET) inhibitors, namely JQ1 and OTX-015. Our studies showed that ATL cell lines were generally sensitive to MYC pathway inhibition with either BET inhibitors (Fig. 3a/b). KOB, ED-40515(−), Tl-Om1 and MT4 cells were consistently the most sensitive cells with a 50% inhibition in cellular proliferation for concentrations in the 0.025 to 0.5 μM range. KK1 and ATL43T were less sensitive to OTX-015 and MT1 cells were less sensitive to JQ1 (Fig. 3a and b). The reasons for these differences are currently unknown but are not associated with genetic mutations in FBXW7. Since BET/BRD inhibition results in the loss of transcription of c-MYC [29, 30], allowing FBXW7-mediated depletion and interruption of the positive auto-regulatory loop, we next wanted to examine the effect of FBXW7 mutations on BET inhibitor’s sensitivity. To this end, FLAG-tagged, wild type FBXW7 and FBXW7 W425R, L443F, S462P and H468R were cloned into a TET-inducible lentiviral vector (pTRIPZ) and high titer pseudotype virus was used to deliver these mutants into MT1 cells (containing a wild type WD40 domain sequence) for puromycin selection. After 3 weeks of selection, successful stable transfer of various FBXW7 mutants was confirmed by adding doxycycline to the culture media and western blot analyses using FLAG antibodies. As expected, adding wild type FBXW7 to MT1 did not alter the sensitivity to JQ1 or OTX-015 despite reduced expression in c-MYC (Fig. 3a and b). In contrast, expression of FBXW7 mutants W425R, S462P and H468R significantly increased the resistance to JQ1 or OTX-015 in short term culture assays (Fig. 3a and b). FBXW7 mutant L443F had an intermediate phenotype consistent with its ability to stimulate partial degradation of c-MYC (see Fig. 2d). The effects of JQ1 and OTX-015 were tested on MT1 cells carrying FBXW7 wild type or S462P mutant over a period of 16 days. In these experimental conditions cell proliferation assays clearly confirmed resistance to both JQ1 and OTX-015; and demonstrated growth advantages of MT1 cells carrying FBXW7 S462P mutation. To demonstrate the specificity of our results and the reliance on MYC signaling, we next used AG-490, a specific JAK2 inhibitor, known to inhibit ATL cell proliferation. As expected, ATL cells were sensitive to treatment with AG-490 (Fig. 3c); however, addition of FBXW7 wild type had no effect and AG-490 did not affect c-MYC expression (Fig. 3c). Consistent with the fact that AG-490 targets the JAK/STAT pathway, FBXW7 mutants did not provide any resistance to this drug (Fig. 3c). Together our data suggest that BET inhibitors may provide limited clinical benefits to patients carrying mutated FBXW7 gene unable to trigger c-MYC degradation and warrant further investigation. Loss of FBXW7 has previously been linked to higher levels of MCL-1 and increased sensitivity to Sorafenib [22]. We then examined the effect of Sorafenib, a multi-kinase inhibitor resulting in loss of MCL-1 expression, and Dinaciclib, a CDK inhibitor shown to block the activity of the cyclin E-CDK2 complex [31]. Both MCL1 and Cyclin E have been shown to be important for HTLV-I transformed cells and ATL cells [32, 33]. To further evaluate the effects of these drug we performed dose dependent cellular proliferation assays. Our results indicated that ATL cell lines were, for the most part, not sensitive to either drug (Fig. 3d and e). Our results also showed that mutations in FBXW7 provided limited (Sorafenib) or no (Dinaciclib) growth advantage when compared to wild type FBXW7 (Fig. 3d and e).

### Increased RAS-RAF signaling causes resistance to BET inhibitors in ATL cells

**Fig. 4:**
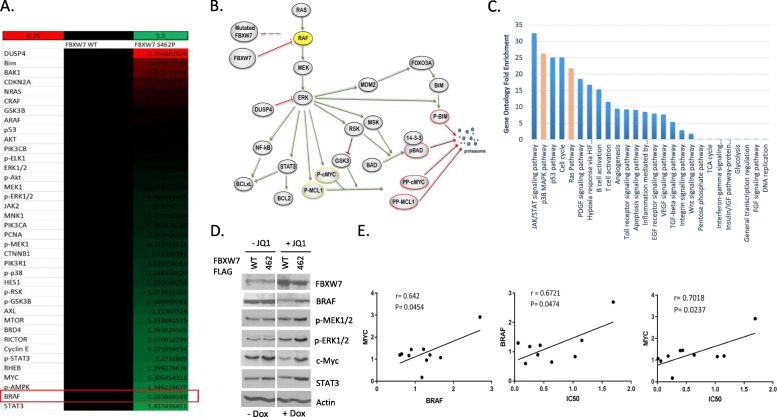
Resistance to BET inhibitors is linked to activation of RAF/MEK/ERK signaling pathway by FBXW7 mutants. **a** RPPA was used to comprehensively analyze the protein expression in MT1 cells expressing FBXW7 WT and S462P. BRAF is up-regulated in MT1 FBXW7 S462P expressing cells compared to MT1 cells expressing FBXW7 WT. **b** Cell signaling pathways affected by wild-type or mutant FBXW7 S462P. (**c**) Gene ontology fold enrichment from MT1 cells expressing FBXW7 WT, S462P, R465C, R505C and R479Q. **d** MT1 cells stably expressing doxycycline inducible FBXW7 WT or S462P mutant were treated with DMSO or JQ1 in the presence or absence of Dox and analyzed for expression of BRAF, p-MEK, p-ERK, c-MYC and STAT3 by Western blot using actin as a loading control. **e** The expression of Myc and BRAF in HTLV-I/ATL-lines was analyzed by WB. Correlation between Myc/BRAF expression and IC50 was calculated by Pearson correlation calculations. JQ1 IC50 in relation to c-MYC or BRAF expression was calculated in ATL-lines treated with different concentration of JQ1 or dimethyl sulfoxide (DMSO) for 72 h. Each cell line was treated at least twice for standard deviation. Cell proliferation was analyzed by XTT assay (Roche)

BET inhibitor JQ1 is known to down-regulate the transcription and expression of c-MYC. However, not all c-MYC-overexpressing cell lines are sensitive to JQ1, suggesting that additional pathways are involved in determining the sensitivity to JQ1 [34]. We next investigated cellular signaling pathways that may be activated/suppressed by mutations in FBXW7 and thereby promote resistance to BET inhibitors JQ1 and OTX-015. To this end cellular lysates from MT1 cells carrying either wild type FBXW7 or the S462P mutation, untreated and treated with JQ1, were analyzed by a high-throughput reverse phase protein array (RPPA). Heat map graphically shows expression changes for 40 proteins with more than 20% difference (from a list of 400 antibodies) (Fig. 4a). Analyses revealed that the RAF-MEK-ERK pathway was the most differentially affected when comparing the FBXW7 wild type and FBXW7 S462P mutant. Downstream targets of ERK were significantly increased such as STAT3, MYC, and RSK while the negative regulator of ERK, DUSP4, was the most repressed gene in our RPPA experiments (Fig. 4a and b). Gene ontology analyses confirmed activation of the RAS/RAF pathway in cells carrying FBXW7 S462P mutations (Fig. 4c). In addition, RAS downstream p38 MAPK signaling and STAT3 were also significantly affected (Fig. 4c). Consistent with the effects of FBXW7 on c-MYC, CyclinE and p53, cell cycle was also significantly affected. RPPA validation was also performed by immunoblots. Addition of doxycycline to the culture media effectively stimulated the expression of wild type or mutated S462P FBXW7 in MT1 cells (Fig. 4d). Consistent with RPPA data and activation of the RAS-RAF pathway, we found increased expression of BRAF, p-MEK, p-ERK, c-MYC and STAT3 in MT1 cells expressing mutated FBXW7 (Fig. 4d). RPPA was used to comprehensively analyze the protein expression in MT1 cells expressing FBXW7 WT, S462P, R465C, R505C and R479Q and a correlation was found between BRAF or c-MYC expression (Fig. 4e).

### BRAF is a novel target of FBXW7 differentially degraded by hot spot mutant R505C; and blockade of BRAF/ERK signaling prevents resistance of ATL cells to BET inhibitor0073

**Fig. 5:**
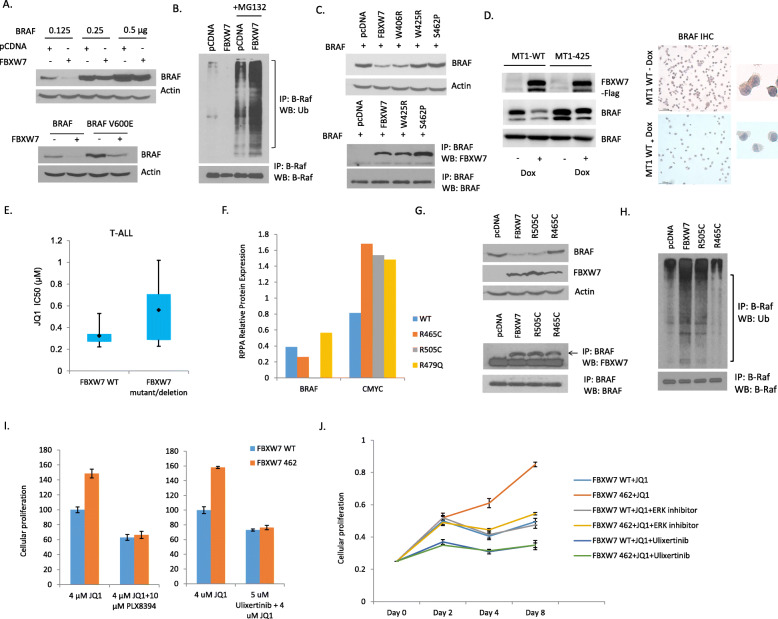
FBXW7 wild type and mutants selectively target BRAF for proteasome degradation. **a** 293 T cells were co-transfected with FBXW7 and increasing amounts of BRAF for 48 h. FBXW7-mediated degradation of BRAF was then determined by Western blot. 293 T cells were co-transfected with FBXW7 and BRAF or BRAF V600E mutant for 48 h. FBXW7-mediated degradation of BRAF or BRAF V600E was determined by Western blot. **b** FBXW7-mediated ubiquitination of BRAF. 293 T cells were co-transfected with FBXW7, K48-Ub and BRAF. Cells were treated with MG132 for 6 h before harvest. Immunoprecipitated BRAF and Western blot Ub showed the ubiquitination level of BRAF. **c** Comparison of FBXW7 wild type and mutants W406R, W425R and S462P in their ability to target BRAF for degradation following transient transfection in 293 T cells. The interaction between FBXW7 WT/mutants and BRAF was analyzed by co-immunoprecipitation. 293 T cells co-transfected with wild-type or mutant FBXW7 and BRAF were lysed and immunoprecipitated with BRAF. Western blot was used to analyze the interaction between FBXW7 WT/mutants and BRAF. **d** ATL cells MT1 stably transfected with a TET-inducible FBXW7 wild type or W425R were induced with doxycycline and degradation (WT) or lack thereof (W425R) of endogenous BRAF was confirmed by Western blot. Endogenous degradation of BRAF by wild type FBXW7 was confirmed by immunohistochemistry following induction of FBXW7 with doxycycline. **e** Graphical representation showing the correlation between FBXW7 gene alternation in T-ALL cell lines and JQ1 IC 50. **f** Data from RPPA was used to quantify BRAF and c-MYC protein expression in MT1 cells expressing FBXW7 WT, R465C, R505C and R479Q. **g** FBXW7-mediated degradation of BRAF by wild type FBXW7 and R505C but not R465C was confirmed in transient transfection assays and Western blot analyses. **h** FBXW7 R505C retains wild type ability to add ubiquitin to BRAF. 293 T cells were co-transfected with FBXW7, K48-Ub and BRAF. Cells were treated with MG132 for 6 h before harvest. Immunoprecipitated BRAF and Western blot Ub showed the ubiquitination level of BRAF. **i** Combination therapy with Ulixertinib or PLX8394 prevents tumor cell resistance to BET inhibitors. MT1 cells carrying an inducible wild type FBXW7 or S462P mutant were cultured in presence of doxycycline and in the presence of JQ1 or JQ1 in combination with BRAF inhibitor PLX8394 or ERK inhibitor Ulixertinib. After 48 h hours cells were collected, and cellular proliferation measured by XTT assays. FBXW7 WT cells treated with JQ1 alone was set as 100% reference. **j** Following the same settings as in (**i**), cellular proliferation was measured by XTT assays every 2 days for 8 days and Doxycycline was added to the media every 2 days

The results presented in Fig. 4b suggest that B-RAF is a target of FBXW7. To confirm these data 293 T cells were transfected with increasing amounts of BRAF in the absence or the presence of an FBXW7 expression vector. As shown in Fig. 5a, FBXW7 was able to stimulate proteasome degradation of BRAF as well as the BRAF mutant V600E. Consistent with these data co-immunoprecipitation assays confirmed that FBXW7 was able to stimulate the ubiquitination of BRAF in transient transfection assays (Fig. 5b). We next tested FBXW7 mutants found in ATL cells characterized in Fig. 2. These studies showed that similar to wild type FBXW7 both W425R and S462P mutants were able to specifically immunoprecipitate BRAF; however, neither mutant was able to stimulate the degradation of BRAF (Fig. 5c). To analyze the interaction and degradation under physiological conditions, the expression of BRAF was analyzed using a stable cell line MT1 cells carrying a TET-inducible wild type or W425R FBXW7. Forty-eight hours after induction with doxycycline endogenous levels of BRAF were significantly decreased in wild type FBXW7 expressing cells but, consistent with transient transfection assays, MT1 cells with the W425R FBXW7 mutant were not able to downregulate endogenous BRAF expression (Fig. 5d). These data were further confirmed using immunohistochemistry detection of endogenous BRAF in MT1 cells carrying a TET-inducible wild type FBXW7 vector. As shown in Fig. 5d induction of FBXW7 expression in the presence of doxycycline significantly reduced expression of BRAF.

FBXW7 has been found to be frequently mutated in T-cell acute lymphocytic leukemia (T-ALL) disease and loss of function of FBXW7 correlate with a higher IC50 following treatment with JQ1 (Fig. 5e). Mutations at R465, R479, or R505 have been shown to disrupt the interaction of FBXW7 with its substrates and to prevent ubiquitin-mediated degradation of FBXW7 substrates [35]. We used RPPA results to comprehensively analyze the expression of BRAF in cells expressing FBXW7 WT, R465C, R505C or R479Q. Consistent with previous studies FBXW7 mutants R465C, R505C or R479Q were unable to degrade c-MYC (Fig. 5f). However, only R465C and R479Q mutants were unable to degrade BRAF while the R505C mutant appeared to behave relatively similar to wild type FBXW7 (Fig. 5f). Since BRAF has not been previously tested, we next tested the ability of R505 and R465 to interact with and promote BRAF degradation. Unexpectedly, R505 and R465 demonstrated different phenotypes. While both mutants were able to bind BRAF, only wild type and R505 were able to promote BRAF degradation (Fig. 5g). These data were further confirmed by ubiquitination assays. Again, R505 and wild type, but not R465, were able to stimulate ubiquitination of BRAF (Fig. 5h). Importantly, these results demonstrate for the first time a phenotypical difference of the hot spot R505C mutant and challenge the previously accepted notion that an Arg mutation at amino acid residue 505 completely inactivates FBXW7 functions.

The lack of BRAF degradation by FBXW7 mutants may result in higher activation of the MEK/ERK pathway leading to an increased phosphorylation and activation of c-MYC. In turn, this coupled with a lack of degradation by FBXW7 mutants W425R, S462P and H468R in part explain lower sensitivity to BET inhibitors. We hypothesized that if resistance to JQ1 provided by FBXW7 S462P occurs through BRAF signaling then specific inhibition of BRAF or the downstream ERK/MEK pathway should impede tumor growth advantage. To test this hypothesis MT1 cells carrying wild type or mutated FBXW7 S462P were treated with JQ1 alone or in combination with BRAF inhibitor PLX8394 or ERK inhibitor Ulixertinib [36, 37]. Cellular proliferation was monitored by XTT assays. While FBXW7 S462P provided a clear growth advantage in the presence of JQ1 this was no longer detectable (Fig. 5i). Long term proliferation assays also confirmed that combination therapy using BET inhibitor along with either BRAF or ERK signaling efficiently prevented growth of tumor cells carrying mutation of FBXW7 (Fig. 5j).

## Discussion

Dimerization of FBXW7 increases its trans-auto-ubiquitination and the turnover of FBXW7 protein. FBXW7 can act as a monomer or a dimer, however dimerization is important for binding of protein with a weak CPD such as cyclin E. In cancer cells FBXW7 mutations are often heterozygote, which would allow the formation of homodimers for both wild type and mutated forms as well as heterodimers. While heterodimers have been shown to be dominant negative for NOTCH1 a complete characterization of FBXW7 targets has not been investigated. Previous studies suggested that mutation in FBXW7 either has a limited effect or possesses a completely inactivate interaction with its targets. Our results challenge this notion and demonstrate that mutations in FBXW7 can selectively disrupt ubiquitination and proteasome degradation of target proteins. We think that lack of degradation of specific targets by FBXW7 may be explained by conformational changes imposed by the mutation on FBXW7. Consistent with this notion, we mutagenized mutant L443F to L443A, L443G and L443H. While the latter did not alter L443F phenotype, L443A regained wild type properties and L443G was deficient only for NCID degradation. We believe that our studies demonstrate a degree of sensitivity in FBXW7 targets. NOTCH1 appear to be the least flexible target and the most frequently lost target upon mutation of FBXW7. We think these data suggest that FBXW7 in its natural conformation and in its mutated form is a druggable target for specific inactivation of downstream signaling pathways and warrants additional studies. Hotspot mutations in FBXW7 have been reported for R505, R465 and R479, and represent 25.41, 9.29 and 13.40%, respectively, of all FBXW7 mutations in cancer [2]. To this date it was generally accepted that these mutations inactivate FBXW7. Our studies challenge this notion and suggest that it may not be the case. We demonstrate here that FBXW7 R505C retains wild type FBXW7 ability to interact with and target BRAF for degradation but was unable to degrade c-MYC, CyclinE or MCL1. As the number of FBXW7 targets continue to be unveiled, we think that for each mutant a clear and distinct pattern of proteins and signaling pathways affected will emerge offering specific therapeutic intervention.

Studies suggest that c-MYC expression is higher in acute ATL patients’ samples and correlates with disease progression. Our study confirms high levels of both c-MYC and MYCN in freshly uncultured ATL samples. Consistent with the fact that c-MYC/MYCN binds to the promoter of microRNA, miR-17-92, which in turn targets PTEN, RNA expression of PTEN was decreased in ATL samples (Fig. 1a). These data contrast with previous findings that in ATL cells PTEN protein levels were similar to normal PBMCs, thus suggesting that post transcriptional stabilization of PTEN occurs in ATL cells. Our studies revealed MYCN as highly upregulated in ATL cells. High expression of MYCN is usually associated with highly proliferative potential of tumor cells and dismal prognosis. Findings in T-ALL suggest that TAL1 pathway activation may sustain the up-regulation of MYCN, whether this is the case in ATL remain to be seen. Interestingly, EZH2 is a target of MYCN and knockdown of MYCN has been found to inhibit the expression of EZH2. Previous studies indicated that EZH2 is not frequently mutated in ATL cells, but it is highly expressed and inversely correlates with H3K27me3 targets. Consistently, the use of an EZH1/2 dual inhibitor reduced ATL cells survival. Observations made with BET inhibitors JQ1 or OTX015 targeting both c-MYC and MYCN warrant additional studies using MYCN specific inhibition such as HUWE1 inhibitors or specific knockdown to differentiate the relative importance of c-MYC and MYCN in ATL tumor cells survival and proliferation.

BET inhibitor JQ1 promotes tumor regression in patient-derived xenografts in vivo and is highly effective in a number of hematological malignancies, including acute myeloid leukemia and multiple myeloma [38]. Although suppression of MYC expression has been demonstrated as a mechanism of growth suppression via BET inhibitors, additional mechanism of action have been reported [39, 40]. Full characterization of mechanisms governing BET inhibitor sensitivity or resistance is of high clinical relevance. Our studies suggest that mutation of FBXW7 may confer resistance of tumor cells to BET inhibitors JQ1 and OTX-015 and at least some mutations within the WD40 domain of FBXW7 provide resistance because of their inability to target BRAF for degradation. Accumulation of BRAF may then activate the MEK-ERK signaling pathway and results in STAT3, c-MYC and MCL1 phosphorylation. In the absence of active GSK3β in ATL cells this would result in stabilization rather than degradation of c-MYC and MCL1. Our studies also suggest that FBXW7 mutation may led to a drastic reduction in DUSP4 expression, a known inhibitor of ERK signaling. In fact, it is likely that ERK signaling in ATL cells plays a central role by inactivation of GSK3β by RSK while simultaneously targeting pro apoptotic BAD for degradation by dual phosphorylation mediated by RSK and MSK (Fig. 4b). Mutation co-occurrences studies found as much as 47% of FBXW7 mutant cells harbor APC mutations, 35% for KRAS and 17% for β-catenin [2]. Oncogenic KRAS promotes activation of the Wnt/β-catenin pathway through LRP6 signaling. These observations suggest that ATL tumor cells with mutated FBXW7 are more likely to display constitutive activation of the KRAS-Wnt/β-catenin signaling axis. In fact, a number of ATL patient cells express LRP6, and over-express LRP5 and Wnt5a, with an active Wnt/β-catenin pathway [41]. Thus, it would be reasonable to assume that these cells may be more dependent than wild type carrying FBXW7 cells upon activation of the Wnt/β-catenin pathway.

## Conclusion

To our knowledge this is the first report to identify FBXW7 mutants with distinct phenotypes. These mutants will be valuable to study the role of specific FBXW7-targeted oncoproteins in cancer progression and drug therapy resistance.

## Abbreviations

HTLV-I: Human T cell Leukemia Virus type 1
ATL: Adult T cell leukemia
FBXW7: F-box/WD repeat-containing protein 7
HAM/TSP: HTLV-1-associated myelopathy/tropical spastic paraparesis
CRC: Colorectal cancer
BET: Bromodomain and Extra-Terminal motif
Tet: Tetracycline
MALM1: Mastermind-like protein 1
FBS: Fetal bovine serum
DMEM: Dulbecco’s Modified Eagle Medium
RPMI: Roswell Park Memorial Institute
RPPA: Reverse-phase protein array
HRP: Horseradish peroxidase
CHX: Cyclohexamide
PCR: Ploymerase chain reaction
IHC: Immunohistochemistry
NF-κB: Nuclear factor kappa-light-chain-enhancer of activated B cells
